# The first complete mitochondrial genome of *Biotodoma cupido* (Cichiliformes: Cichlidae) and its phylogeny

**DOI:** 10.3389/fgene.2025.1623517

**Published:** 2025-08-29

**Authors:** Xiaoli Zhang, Shuang-Xi Jia, Cheng-He Sun

**Affiliations:** ^1^ College of Environment and Life Health, Anhui Vocational and Technical College, Hefei, China; ^2^ College of Life Sciences, Nanjing Forestry University, Nanjing, China

**Keywords:** Biotodoma cupido, cichlidae fish species, hybridization, mitochondrial genome, phylogenetic analysis

## Abstract

Traditional classifications of New World cichlids have been subject to persistent controversy. Within the genus *Biotodoma*, only two species are currently recognized; however, complete mitochondrial sequences for these taxa have remained unavailable. In the present study, we sequenced and characterized the complete mitochondrial genome of *Biotodoma cupido.* This mitogenome has a total length of 16,621 bp and encodes the standard 37 genes found in vertebrate mitochondria: 13 protein-coding genes (PCGs), 22 transfer RNA (tRNA) genes, two ribosomal RNA (rRNA) genes, and one non-coding control region (D-loop). Among the PCGs, only Cox1 gene utilizes GTG as its start codon, while the remaining 12 PCGs start with ATG. Observed termination codons included TAA, AGA, TAG, and the incomplete codons TA and T. The overall base composition of the *B. cupido* mitochondrial sequence exhibits an A + T bias, with a combined A + T content of 54.1%. In this study, the high mitogenome similarity observed among several species in this study resulted from interspecific hybridization rather than synonymy or taxonomic misidentification. Maximum likelihood and Bayesian inference evolutionary trees were constructed using mitochondrial genome sequences from 44 Cichlidae species. Phylogenetic analyses consistently recovered the tribes Geophaginae, Cichlasomatinae, and Cichlinae as monophyletic groups. In contrast, the tribe Astronotinae was recovered as polyphyletic. These results clarify the evolutionary position of *B. cupido* within New World cichlids and will contribute to elucidating the complex phylogenetic relationships among cichlid species.

## 1 Introduction

*Biotodoma cupido* (Heckel, 1840) belongs to the order Cichliformes and the family Cichlidae. Its distribution encompasses much of the Amazon Basin, extending from the Ucayali River system in Peru eastwards through the Brazilian Amazon to the Tocantins drainage, which flows into the Atlantic Ocean alongside the Amazon Delta. The southern limit of its range appears to be the *Río* Mamoré in Bolivia, a tributary of the *Rio* Guaporé, and the northern limit is the Essequibo basin, Guyana (Cichocki, 1977; Morey et al., 2019; Morey et al., 2024). This South American cichlid, reaching approximately 15 cm in length, is valued as an ornamental species. *B*. *cupido* exhibits a mild temperament and omnivorous feeding habits, consuming water worms, snails, minced meat, and artificial feed. While females and males attain similar body lengths, males display more vibrant coloration. Upon reaching sexual maturity, males develop a pronounced nuchal hump and large body size, with distinctive black spots on their dorsal and anal fins (Morey et al., 2024).

Molecular methods have become indispensable tools in fish classification and phylogenetic studies (Wang et al., 2023). This prominence stems primarily from the inherent stability of genetic information, which remains largely unaffected by environmental factors. Furthermore, modern sequencing technologies enable quantitative evolutionary analyses, thereby elucidating evolutionary mechanisms. Mitochondrial DNA (mtDNA) polymorphisms are particularly valuable as they resist environmental influences and directly reflect interspecific genetic relationships. Leveraging these unique characteristics - such as relatively high mutation rates, absence of introns, and predominantly maternal inheritance - mtDNA-based molecular phylogenetic analysis has evolved into a robust and reliable methodology.It is now widely applied across diverse fields including evolutionary genomics, systematics, and molecular evolution studies. (Ki et al., 2009). In fish, several partial mitochondrial sequences such as Cyt*b*, COI have been utilized for species identification (Mattos and Costa, 2018; Ottoni and Mattos, 2015), phylogenetic analysis (Lopez-Fernández et al., 2010; Musilová et al., 2008; Concheiro-Pérez et al., 2007) and species diversification analysis (Kullander et al., 2010; Piálek et al., 2012; Tougard et al., 2017). For a phylogenetic relationship multiple gene analysis is more powerful than analysis using single markers (Blanco-Bercial et al., 2011). Additionally, the abundance of mtDNA polymorphism serves as a crucial tool for species group identification (Ye et al., 2023). Comparative analyses of mtDNA sequences across populations, when combined with biological data, form the foundation for investigating fish population genetics (Reiss et al., 2009).

Research by taxonomists suggests that, in terms of morphological characteristics, *B. cupido* represents a basal offshoot of the phyletic lineage culminating in the large genera *Apistogramma* and *Geophagus* (Cichocki, 1976). Species of this type often retain the most ancestral morphological traits and carry a greater number of ancestral gene clusters. Consequently, they can provide an evolutionary “reference framework” for reconstructing the ancestral trait states, which holds significant evolutionary importance and biological implications. Although *B. cupido* has not yet been listed in the IUCN Red List of Threatened Species by the International Union for Conservation of Nature, basal offshoot species with unique genetic reservoirs are considered to have high priority for ecological conservation.

To date, only two species (*B*.*cupido* and *Biotodoma wavrini*) of the genus *Biotodoma* (Leibel, 1995) have been reported, and complete mitochondrial sequences are currently unavailable for both species. In this study, we report the complete mitogenome structure and characteristics of *B. cupido*. Furthermore, we conducted phylogenetic reconstruction using the newly generated B. cupido mitogenome alongside other available complete Neotropical cichlid mitochondrial genomes sourced from GenBank, to rigorously evaluate its phylogenetic position. Such a comparative analysis of whole mitogenomes provided new insights into the evolutionary relationships in New World cichlids and reaffirmed the phylogenetic position of *B. cupido*.

## 2 Materials and methods

### 2.1 Sample collection and DNA extraction

All procedures were conducted in accordance with relevant ethical guidelines. Specimen collection complied with applicable legislation in China and followed protocols approved by the Animal Ethics Committee of Nanjing Forestry University. All experimental protocols adhered to international standards for animal welfare, including the Convention on Biological Diversity (CBD), the Nagoya Protocol, and the ARRIVE guidelines (https://arriveguidelines.org). Samples (in *vitro*-bred ornamental fish of Chinese origin) were collected on 1 April 2022 from an aquatic market in Qinhuai District, Nanjing City, Jiangsu Province (32.005899°N, 118.841977°E). Tail fin tissues were immediately immersed in anhydrous ethanol and stored at −20°C. Genomic DNA was extracted from the samples using the CTAB method (Porebski et al., 1997). The integrity of the extracted DNA was assessed by agarose gel electrophoresis, while its purity was determined through spectrophotometric analysis.

### 2.2 Library construction and sequencing

Genomic DNA was fragmented using a Covaris ultrasonicator (Covaris, USA) to generate randomly sheared fragments. Subsequently, PCR amplification and other standard procedures were performed for library preparation. The constructed library was diluted following preliminary quantification with a Qubit 3.0 Fluorometer (Nakayama et al., 2016), and the insert size distribution was determined using a Qsep100 Bioanalyzer (BiOptic Inc., New Taipei, Taiwan). Quantitative PCR (qPCR) was conducted to precisely measure the library’s effective concentration (with a threshold of >3 nM). Upon passing quality control, libraries were pooled at appropriate ratios based on their effective concentrations and the required sequencing depth. Following DNA library qualification, paired-end sequencing was performed on the Illumina HiSeq high-throughput sequencing platform, generating a minimum of 10 Gb of raw data.

### 2.3 Sequence assembly

Raw sequencing data underwent quality control using FastQC, followed by adapter trimming and quality filtering with Trimmomatic. This process retained >90% of reads, yielding trimmed sequences averaging ∼140 bp in length. The average sequencing depth exceeded 20× coverage. All library preparation and quality assessments were performed by Nanjing Qingke Biotechnology Co., Ltd. (Nanjing, China). Upon receiving the raw sequencing data from the company, we employed NOVOPlasty for sequence assembly and optimization (Dierckxsens et al., 2017). The pipeline consisted of the following steps: (1) using BLAST to identify complete mitochondrial genome sequences with high similarity to our input sequences; (2) locating and modifying the config.txt file in the NOVOPlasty directory by adjusting parameters including *Type*, *Genome Range*, and *Reference sequence* (default parameters were used in NOVOPlasty); (3) generating of complete mitochondrial genome sequence in FASTA format; (4) performing preliminary genome annotation through the MITOS web server (Bernt et al., 2013); (5) selecting reference sequences showing both high similarity and close phylogenetic relationships using BLAST; and (6) validating the MITOS preliminary annotations using selected reference sequences as benchmarks. Transfer RNA (tRNA) genes were identified using both MITOS2 and tRNAscan-SE v2.0. (Lowe and Eddy, 1997). Ribosomal RNA (rRNA) localization proved more challenging and was primarily inferred based on their conserved positional relationships with flanking genes.

### 2.4 Bioinformatic analysis

We analyzed the base composition and codon usage frequency of the annotated mitochondrial genome sequence using MEGA software (version 11) (Kumar et al., 1994). The AT-skew and GC-skew values were calculated according to the following formulas: AT-skew = (A−T)/(A + T) and GC-skew = (G−C)/(G + C) (Perna and Kocher, 1995). Finally, we generated a circular representation of the complete mitochondrial genome using the MitoFish web server (Zhu et al., 2023).

### 2.5 Phylogenetic analysis

The assembled mitochondrial genome of *B. cupido*, together with 42 New World cichlid mitogenomes from GenBank, was included in the phylogenetic analysis. An African cichlid mitogenome served as the outgroup. A total of 44 mitochondrial genomes were selected for phylogenetic analysis based on their mitochondrial protein-coding gene (CDS) sequences (Table 1). These 44 species mitogenomes were processed using PhyloSuite v1.2.3 (Zhang et al., 2020). Sequences were initially aligned with MAFFT (Katoh and Standley, 2013) using codon-based alignment, followed by optimization in MACSE (Ranwez et al., 2018). The concatenated dataset comprised 13 CDS sequences from all 44 species. This combined dataset was subsequently analyzed with the ModelFinder plugin for optimal data partitioning and gene-specific model selection (Kalyaanamoorthy et al., 2017). For phylogenetic reconstruction, we employed both Bayesian inference (BI) and maximum likelihood (ML) methods through the MrBayes (Ronquist et al., 2012) and IQ-TREE (Minh et al., 2020) plugins, respectively. The best-fit models selected were GTR + F + I + G4 for BI analysis and GTR + F + R5 for ML analysis.

**TABLE 1:** Complete mitochondrial sequence used in this study.

Tribe	Organism	Length (bp)	Accession No
Astronotinae	*Astronotus ocellatus*	16,569	AP009127
Astronotinae	*Chaetobranchopsis bitaeniatus*	16,610	KR150861
Cichlasomatinae	*Aequidens metae*	16,541	KR150865
Cichlasomatinae	*Amphilophus amarillo*	16,521	KY315559
Cichlasomatinae	*Amphilophus citrinellus*	16,522	KJ081546
Cichlasomatinae	*Andinoacara pulcher*	16,513	KR150868
Cichlasomatinae	*Andinoacara rivulatus*	16,585	LC009435
Cichlasomatinae	*Bujurquina mariae*	16,540	KR150862
Cichlasomatinae	*Bujurquina oenolaemus*	16,532	KX397358
Cichlasomatinae	*Cichlasoma dimerus*	16,617	KR150876
Cichlasomatinae	*Cryptoheros cutteri*	16,528	KR150878
Cichlasomatinae	*Herichthys cyanoguttatus*	16,540	KR150867
Cichlasomatinae	*Heros severus*	16,577	MT363636
Cichlasomatinae	*Hypselecara temporalis*	16,544	AP009506
Cichlasomatinae	*Krobia guianensis*	16,539	KR233978
Cichlasomatinae	*Laetacara thayeri*	14,315	KR233974
Cichlasomatinae	*Nannacara anomala*	16,502	KU531436
Cichlasomatinae	*Parachromis managuensis*	16,526	KP728467
Cichlasomatinae	*Petenia splendida*	16,518	KJ914664
Cichlasomatinae	*Pterophyllum altum*	16,495	KT180164
Cichlasomatinae	*Pterophyllum scalare*	16,491	KP231206
Cichlasomatinae	*Rocio octofasciata*	16,539	KR150870
Cichlasomatinae	*Symphysodon aequifasciata*	16,545	KT362183
Cichlasomatinae	*Symphysodon discus*	16,544	KP313730
Cichlasomatinae	*Symphysodon haraldi*	16,543	KT215609
Cichlasomatinae	*Thorichthys aureus*	16,530	KU531435
Cichlasomatinae	*Thorichthys meeki*	16,527	MZ427899
Cichlasomatinae	*Uaru amphiacanthoides*	16,549	KR150875
Cichlasomatinae	*Vieja melanura*	16,543	KF879808
Cichlinae	*Cichla monoculus*	16,526	OR601300
Cichlinae	*Cichla ocellaris*	16,526	KU878410
Cichlinae	*Cichla piquiti*	16,536	OR601299
Cichlinae	*Cichla temensis*	16,530	OR601301
Cichlinae	*Crenicichla regani*	11,461	KR233977
Geophaginae	*Apistogramma cacatuoides*	16,870	KR150874
Geophaginae	*Biotodoma cupido*	16,621	OP595705
Geophaginae	*Dicrossus filamentosus*	11,887	KR233975
Geophaginae	*Geophagus brasiliensis*	16,559	KU531434
Geophaginae	*Geophagus steindachneri*	16,594	KR150866
Geophaginae	*Gymnogeophagus balzanii*	16,587	KR150864
Geophaginae	*Mikrogeophagus ramirezi*	16,526	KR233976
Geophaginae	*Taeniacara candidi*	16,581	KR150873
Retroculinae	*Retroculus lapidifer*	16,537	KR150871
Pseudocrenilabrinae	*Copadichromis borleyi*	16,581	OQ558013

Phylogenetic reconstruction using Bayesian inference (BI) was performed on the concatenated dataset of 13 protein-coding genes (CDS) derived from 44 selected species, utilizing the MrBayes plugin within PhyloSuite. The analysis ran two independent Markov chains, with the first 25% of generations discarded as burn-in. We performed 100 million generations in total, sampling every 100 generations. For maximum likelihood (ML) analysis, we employed the IQ-TREE plugin with 50,000 ultrafast bootstrap replicates. The resulting phylogenetic trees were subsequently visualized and annotated using the interactive Tree of Life (iTOL) online platform (Letunic and Bork, 2021).

## 3 Results

### 3.1 Basic structure of B. cupido mitochondrial genome

**FIGURE 1:**
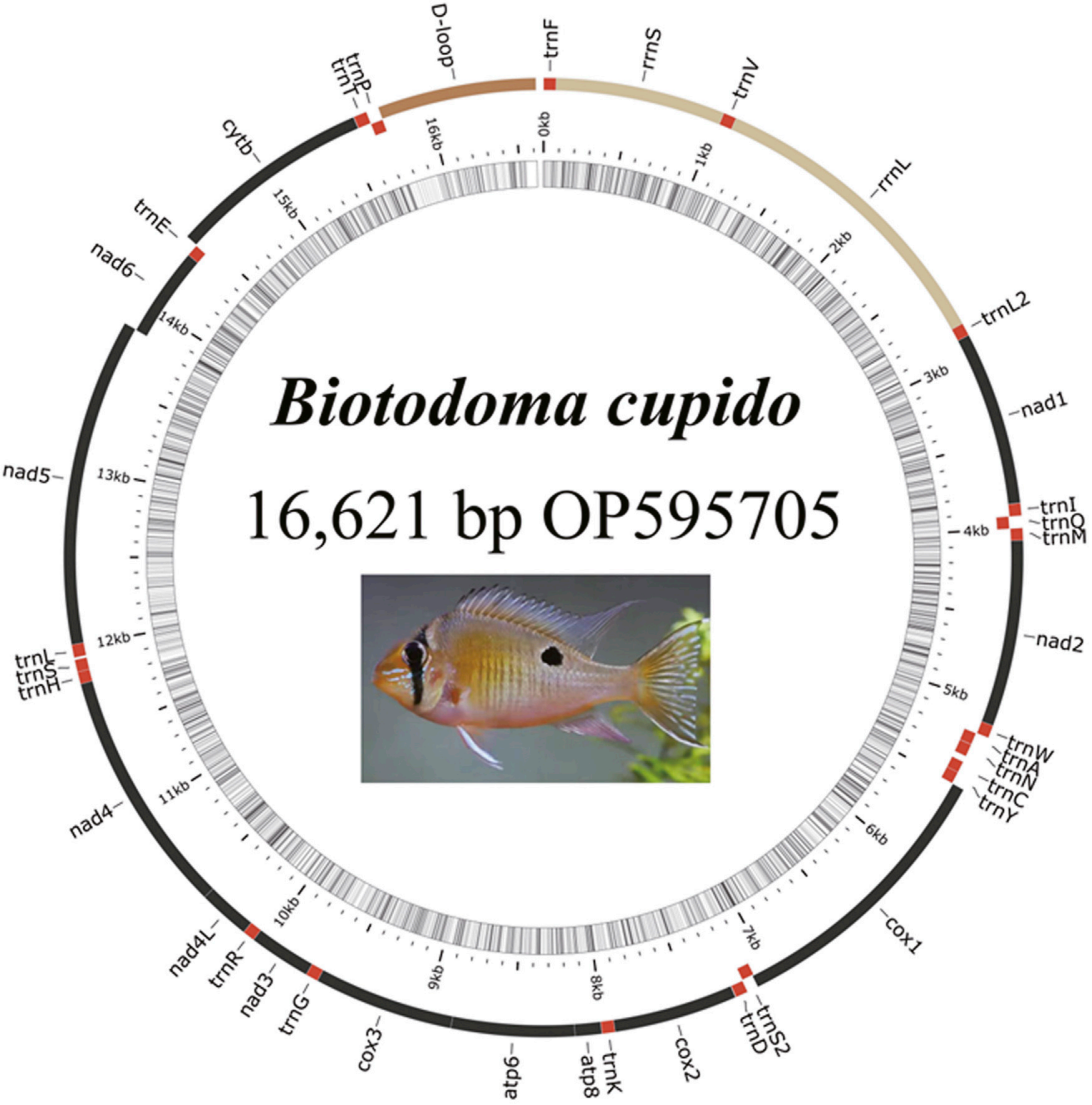
Gene map of the *Biotodoma cupido* mitogenome.

The complete mitochondrial genome of *B. cupido* (GenBank Accession No. OP595705) measured 16,621 bp long, exhibiting a typical circular double-stranded structure (Figure 1; Table 2). This genome contains 37 functional genes and an 891 bp control region. Gene distribution analysis revealed that nine genes were encoded on the light (L) strand, comprising one protein-coding gene (nad6) and eight tRNA genes (trnQ, trnA, trnN, trnC, trnY, trnS2, trnE, and trnP). The remaining 28 genes resided on the heavy (H) strand, including 12 protein-coding genes, 14 tRNAs, and two rRNA genes. The genome organization showed 11 intergenic spacer regions ranging from 1 to 35 bp, with the largest spacer located between trnN and trnC. Additionally, we identified eight overlapping gene regions (1-19bp in length), with the most extensive overlap occurring between atp8 and atp6.

**TABLE 2:** Complete annotation of mitochondrial genome results for *Biotodoma cupido*.

Gene	Position		Size	Intergenic nucleotides	Codon		Strand
	From	To			Start	Stop	
trnF	1	70	70	0			H
rrnS	71	1,022	952	0			H
trnV	1,023	1,095	73	0			H
rrnL	1,096	2,814	1,719	0			H
trnL2	2,815	2,887	73	0			H
nad1	2,888	3,862	975	0	ATG	TAA	H
trnI	3,864	3,933	70	1			H
trnQ	3,932	4,002	71	−2			L
trnM	4,002	4,070	69	−1			H
nad2	4,071	5,115	1,045	0	ATG	T	H
trnW	5,116	5,187	72	0			H
trnA	5,190	5,258	69	2			L
trnN	5,260	5,332	73	1			L
trnC	5,368	5,435	68	35			L
trnY	5,435	5,504	70	−1			L
cox1	5,506	7,104	1,599	1	GTG	AGA	H
trnS2	7,095	7,165	71	−10			L
trnD	7,168	7,237	70	2			H
cox2	7,241	7,934	694	3	ATG	T	H
trnK	7,935	8,007	73	0			H
atp8	8,009	8,176	168	1	ATG	TAA	H
atp6	8,158	8,849	692	−19	ATG	TA	H
cox3	8,850	9,633	784	0	ATG	T	H
trnG	9,634	9,704	71	0			H
nad3	9,705	10,053	349	0	ATG	T	H
trnR	10,054	10,122	69	0			H
nad4L	10,123	10,419	297	0	ATG	TAA	H
nad4	10,413	11,790	1,378	−7	ATG	T	H
trnH	11,791	11,859	69	0			H
trnS	11,860	11,928	69	0			H
trnL	11,941	12,013	73	12			H
nad5	12,014	13,852	1,839	0	ATG	TAA	H
nad6	13,849	14,370	522	−4	ATG	TAG	L
trnE	14,371	14,439	69	0			L
cytb	14,444	15,583	1,140	4	ATG	TAA	H
trnT	15,586	15,660	75	2			H
trnP	15,660	15,730	71	−1			L
D-loop	15,731	16,621	891	0			H

### 3.2 Base composition of B. cupido mitochondrial genome

The base composition of the *B. cupido* mitochondrial genome was analyzed using MEGA11 (Table 3). The overall base frequencies are as follows: A = 27.5%, T = 26.6%, C = 30.9%, and G = 14.9%, demonstrating a significant A + T bias (54.1% combined) relative to G + C content. Skewness values further indicate compositional asymmetry: a positive AT-skew (0.017) suggests a slight preference for adenine (A) over thymine (T), while a negative GC-skew (−0.350) indicats a stronger bias toward cytosine (C) over guanine (G). For protein-coding regions (total length: 11,475 bp), the base composition was A = 25.0%, T = 28.6%, C = 31.8%, and G = 14.9%, maintaining the A + T bias (53.6%). Among codon positions, the second position exhibited the strongest A + T preference (58.3%).

**TABLE 3:** Base composition of the *Biotodoma cupido* mitogenome.

Regions	Size (bp)	T(U)	C	A	G	AT (%)	GC (%)	AT skew	GC skew
Full genome	16,621	26.6	30.9	27.5	14.9	54.1	45.9	0.017	−0.350
CDS	11,475	28.6	31.8	25.0	14.5	53.6	46.4	−0.066	−0.373
1st codon position	3,825	21.1	28.9	26.1	23.9	47.2	52.8	0.106	−0.095
2nd codon position	3,825	40.5	28.2	17.8	13.5	58.3	41.7	−0.390	−0.350
3rd codon position	3,825	24.1	38.5	31.2	6.2	55.3	44.7	0.128	−0.722

### 3.3 CDS and codon usage

**FIGURE 2:**
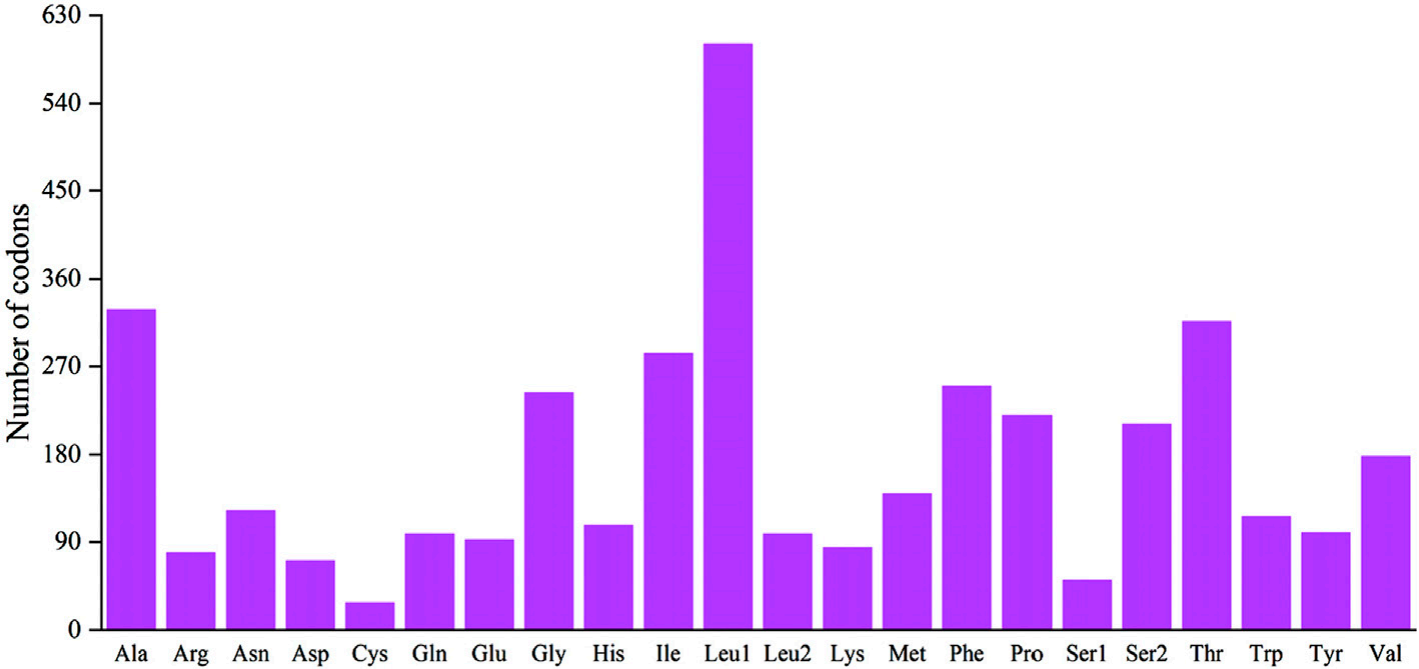
Codon distribution of the *B*. *cupido* mitogenome. Numbers on the Y-axis refer to the total number of codons, and codon families are provided on the X-axis.

**FIGURE 3:**
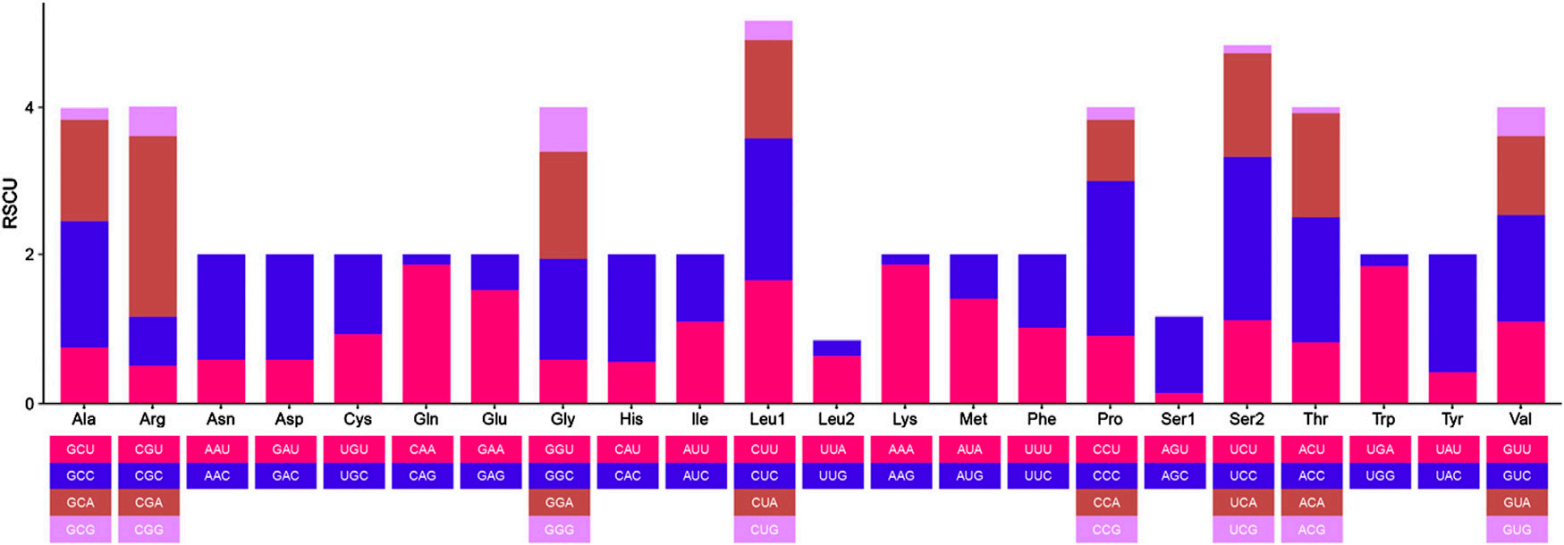
Relative synonymous codon usage (RSCU) of the protein-coding genes (CDS) of *B*. *cupido* mitogenomes.

The 12 CDS in *B. cupido* use the typical ATG as the start codon, whereas *cox1* use GTG as the start codon. Termination codon usage is more variable: five CDS terminate with TAA; *nad6* uses TAG, *cox1* uses AGA and five genes (*nad2*, *cox2*, *cox3*, *nad3*, and *nad4)* end with an incomplete T, while *atp6* terminates with an incomplete TA (Table 2). The incomplete termination codons are completed by the addition of 3′A to the mRNA. MEGA was used to statistically analyze the codon usage and amino acid contents of the sequences (Figures 2, 3). The most frequently noted amino acids were Leu1 (15.74%), Ala (8.62%), Thr (8.30%), Ile (7.41%), and Phe (6.55%), accounting for a total of 46.62%. Cys exhibited the lowest content (0.73%) (Figure 2). The CDS used 3,825 codons, with the most frequently used codon being CUC (224 times), with a relative synonymous codon usage (RSCU) value of 1.92, followed by CUU (192 times), with an RSCU value of 1.65. All codons encoded proteins (Figure 3).

### 3.4 tRNA gene, rRNA gene, and D-loop

The 22 tRNAs genes range in length from 68 to 75 bp. The longest tRNA gene is trnT (75 bp), and the shortest is trnC (68 bp). Among the 22 tRNA genes, *trnQ*, *trnA*, *trnN*, *trnC*, *trnY*, *trnS2*, *trnE*, and *trnP* are determined to be encoded on the light strand, whereas the other 14 tRNA genes are found to be encoded on the heavy strand. The total length of the tRNA genes is 1558 bp. The two rRNA genes, 12S ribosomal RNA (*rrnL)* is located between the *trnV* and *trnL2* tRNAs, with a length of 1719 bp, whereas 16S ribosomal RNA (*rrnS)* is located between *trnF* and *trnV*, with a length of 952 bp. The A + T content of the two rRNA sequences is 52.4%, similar to the A + T content of the entire sequence but only slightly higher than the G + C content. The non-coding region (D-loop) is located between *trnP* and *trnF*, with a length of 891 bp.

### 3.5 Phylogenetic analysis

**FIGURE 4:**
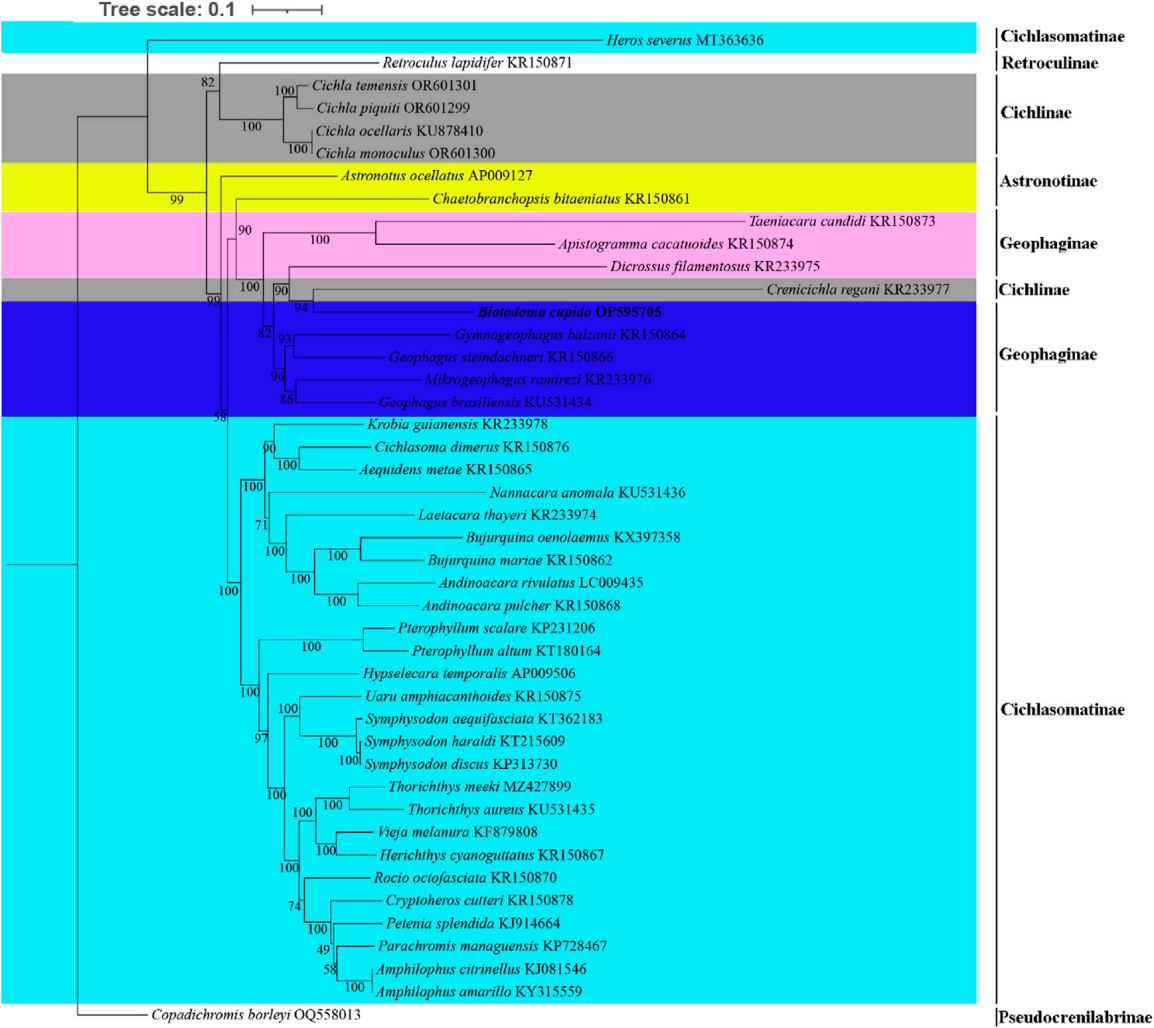
Molecular phylogenetic tree constructed based on maximum likelihood analysis. Numbers at nodes represent the bootstrap support in the ML analysis. In the constructed phylogenetic tree, distinct colors represent different species clusters:cyan for tribe Cichlasomatinae, gray for tribe Cichlinae, pink and dark blue for tribe Geophaginae, and yellow for tribe Astronotinae.

**FIGURE 5:**
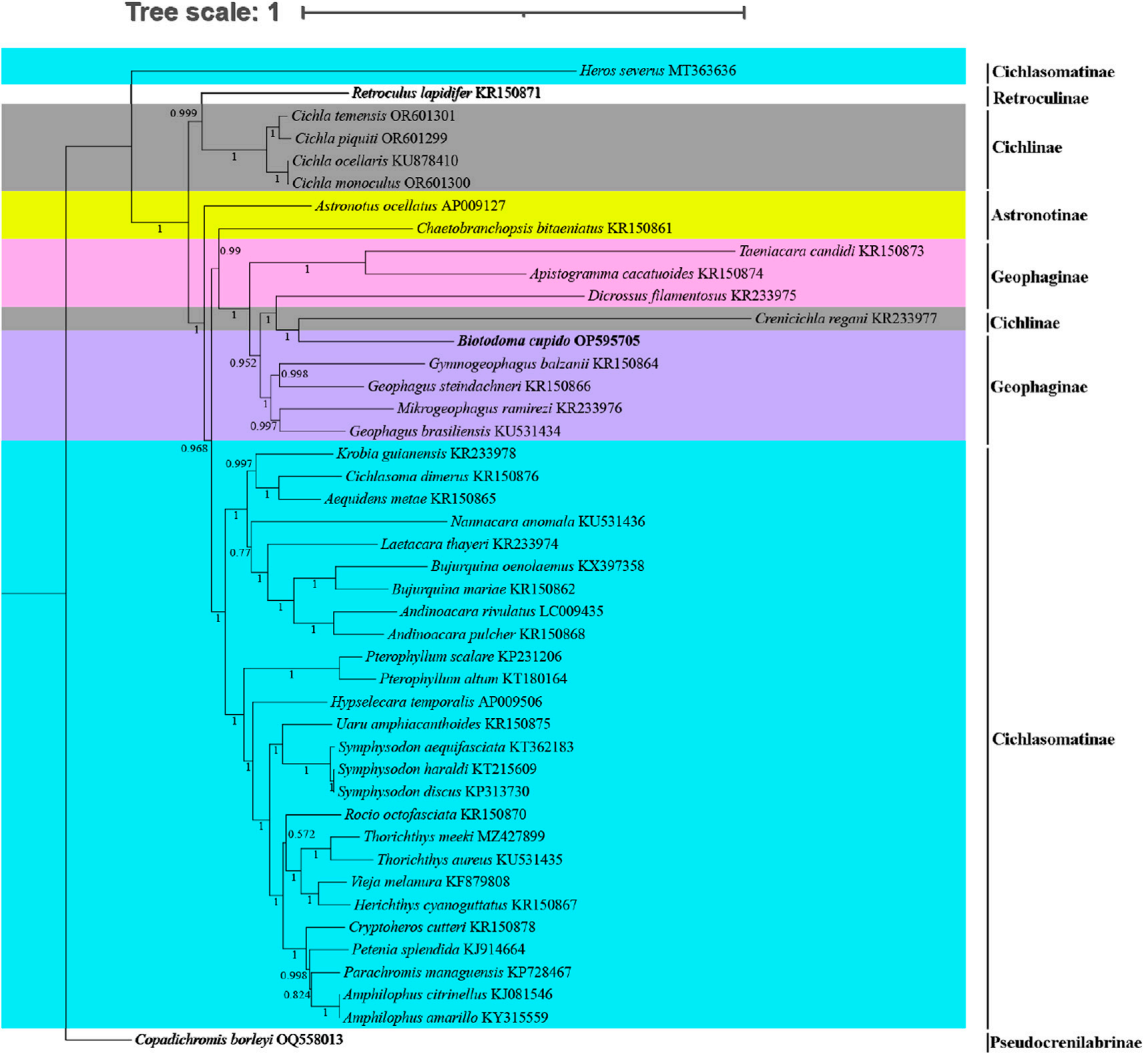
Molecular phylogenetic tree constructed based on Bayesian inference analysis. Numbers at nodes represent the Bayesian posterior probability in the BI analysis. In the constructed phylogenetic tree, distinct colors represent different species clusters:cyan for tribe Cichlasomatinae, gray for tribe Cichlinae, pink and purple for tribe Geophaginae, and yellow for tribe Astronotinae.

For phylogenetic analysis, we selected mitochondrial genome CDS from 43 New World cichlid species representing five tribes (Astronotinae, Cichlasomatinae, Cichlinae, Geophaginae, and Retroculinae), along with one African cichlid species (subfamily Pseudocrenilabrinae) as the outgroup. Both maximum likelihood (ML) and Bayesian inference (BI) trees were constructed using the concatenated sequences of 13 protein-coding genes (Figures 4, 5). Most nodes in these phylogenetic trees showed strong statistical support. The topologies of the ML and BI trees were largely congruent, with minor discrepancies primarily attributed to variations in the phylogenetic placement of *Rocio octofasciata*.

As shown in Figures 4, 5, all 42 New World Cichlids (except for *Heros Severus*) were grouped into a monophyletic group well supported (bootstrap support = 99%; Bayesian posterior probability = 1.0). Among the five New World Cichlid tribes, Geophaginae, Astronotinae, Cichlasomatinae and Cichlinae were found to be polyphyletic; only Retroculinae was monophyletic (represented by a single species and thus not evaluated for monophyly). Both ML analysis and BI analysis supported a sister-group relationship between the tribe Cichlasomatinae (except *Heros severus*) and the tribe Geophaginae (bootstrap support = 58%; Bayesian posterior probability = 0.96). Additionally, both ML analysis and BI analysis supported a sister-group relationship between the tribe Retroculinae and the tribe Cichlinae (except *C*. *regani*) (bootstrap support = 82%; Bayesian posterior probability = 0.99). The phylogenetic analyses also revealed that species within the tribe Cichlini occupied a basal position in the tree, while species from the tribes Cichlasomatini (except *H. severus*) and Geophagini formed a clade at the apex of the evolutionary tree.

The tribe Astronotinae occupied an intermediate evolutionary position between Geophaginae and Cichlinae. Specifically, *Astronotus ocellatus* (Astronotinae) formed a sister group to Cichlinae, whereas *Chaetobranchopsis bitaeniatus* (Astronotinae) exhibited a sister relationship with Geophaginae. Similarly, with the exception of *C. regani*, the phylogenetic analysis indicated that the tribe Geophaginae was monophyletic. Tribe Geophaginae comprised two well-supported clades, both with maximum support values (bootstrap support = 100%; Bayesian posterior probability = 1.00). The first subclade included genera *Gymnogeophagus*, *Geophagus*, *Mikrogeophagus*, *Dicrossus*, *Biotodoma* and *Crenicichla* (from tribe Cichlinae) (bootstrap support = 96%; Bayesian posterior probability = 1.00). The second subclade consisted of genera *Taeniacara* and *Apistogramma* (bootstrap support = 100%; Bayesian posterior probability = 1.00). With the exception of *H. severus*, the phylogenetic analysis also showed that the tribe Cichlasomatinae was monophyletic. All six examined genera within the tribe Cichlasomatinae (*Amphilophus*, *Andinoacara*, *Bujurquina*, *Pterophyllum*, *Symphysodon* and *Thorichthys*) containing two or more species were reciprocally monophyletic*.* Notably, genetic similarity exceeded 99.9% between *Amphilophus amarillo* and *A. citrinellus*, *Symphysodon discus* and *Symphysodon haraldi*, and *Cichla monoculus* and *C. ocellaris*. Phylogenetic relationships of Cichlidae were reconstructed after removing *Crenicichla regani* and *H. severus*. No significant changes were observed in the position of the target species or other taxa in the phylogenetic tree compared to the original topology (see Supplementary Material, Supplementary Figure S1, S2).

## 4 Discussion

The mitochondrial genome of *B. cupido* was found to be similar to those of other Cichlids species in terms of gene quantity and organizational structure (Adrian-Kalchhauser et al., 2017). The mitogenome of *B. cupido* was 16,621 bp long and encoded 37 genes (13 protein-coding genes, two ribosomal RNA genes, 22 transfer RNA genes, and a control region). The AT bias of *B. cupido* (54.1%) was slightly higher than that of *Lamprologus ornatipinnis* and *Coptodon camerunensis* (53.9% and 52.63%), while it was lower than Lamprologus meleagris (55.1%) (Wang et al., 2023; Kundu et al., 2023). 12 CDS in *B. cupido* used the typical ATG as the starting codon, whereas cox1 used GTG as the starting codon, as exhibited in *C. camerunensis* (Kundu et al., 2023). There was no essential difference in the *codon* usage patterns of other Cichlids species. Except for nad6 and 8 tRNAs located on the L chain, the remaining 26 coding genes were located on the H chain, similar to those found in other fish (Yu and Kwak, 2015).

The mitochondrial genome of *B. cupido* contains both rrnL and rrnS single-copy genes with no overlapping intervals, which is consistent with the typical characteristics of metazoans (Gissi et al., 2008). Moreover, the rRNA sequence of *B. cupido* is highly conserved, which is similar to that of other bony fish, based on a BLAST comparison (Jondeung et al., 2007). All 13 CDS in *B. cupido*, the starting codon was similar to that in common bony fish, with two relatively stable starting codons, GTG and ATG. The ending codons were also common among bony fish, and these included TAA, TAG, and incomplete T. In addition, no special codons were used. The 3′end of the transcription product of the incomplete termination codon is U, and mitochondrial mRNA undergoes poly-A modification after transcription to terminate the T codon and ultimately form the UAA termination codon (Bibb et al., 1981).

Lopez-Fernández et al. (2005) established two Geophaginae subclades: one comprising *Geophagus sensu lato*, *Gymnogeophagus*, *Mikrogeophagus*, *Biotodoma*, *Crenicara*, and *Dicrossus*; the other including *Satanoperca*, *Apistogramma*, *Apistogrammoides*, and *Taeniacara*. These well-supported clades align with our findings. Lopez-Fernández et al. (2010) study further demonstrate that Neotropical cichlids (subfamily Cichlinae) are strongly monophyletic. The subfamily Cichlinae includes the tribes Chaetobranchini, Astronotini, Geophagini, Cichlasomatini and Heroini, with the latter being sister to a monophyletic group. For example, in López’s study, both species of *Biotodoma* were recovered as a monophyletic group, which in turn was identified as the sister group to *Crenicara* + *Dicrossus* clade. However, in our study, the result showed that Geophaginae, Astronotinae, Cichlasomatinae and Cichlinae are polyphyletic. Our results indicate that the polyphyly observed in the three tribes (Cichlasomatinae, Cichlinae, and Geophaginae) stemmed from the phylogenetic placement of *H. severus* and *C. regani*. Hybridization leads to morphological features that are discordant with mitochondrial genomic data, thereby causing the phylogenetic relationships of groups to exhibit polyphyly. In this study, the mitochondrial genome data of *H. severus* and *C. regani* are found to contain anomalies. To ensure the integrity and credibility of our study, we use all available mitochondrial genome data of Neotropical cichlid species from the NCBI database when establishing their phylogenetic relationships. For the mitochondrial genome of *C. regani*, the similarities of the COI, 16S rRNA, and ND2 gene fragments compared with other species within the same genus are 99.08%, 98.89%, and 100%, respectively. However, the similarity of the Cytb gene fragment to congeneric species is only 86%. Based on this result, we consider the mitochondrial genome of *C. regan*i (accession no. KR233977) unreliable and should be discarded. For *H. severus*, the mitochondrial gene fragment data showed no match to those of the same species published in the NCBI database. This mismatch is likely attributed to taxonomic misclassification caused by an unresolved taxonomic framework. Based on these result, we prefer to consider the mitochondrial genome of *H. severus* (accession no. MT363636) unreliable and should be discarded.

Following the exclusion of two problematic mitochondrial genome datasets, our results showed that the tribes Geophaginae, Cichlasomatinae, and Cichlinae are monophyletic, while Astronotinae remains polyphyletic. Colatreli et al. (2012) demonstrated profound phylogenetic divergences within the tribe Astronotinae, noting incongruence between morphological traits and genetic differentiation. Their phylogenetic distribution tests of ocelli also indicate a polyphyletic origin. These factors may collectively explain why Astronotinae remains polyphyletic.

Cichlid fishes are well known for spectacular evolutionary radiations, as they have repeatedly evolved into large and phenotypically diverse arrays of species. A study of 412 Cichlid species demonstrated that nucleotide diversity within species is low and the divergence within radiations is also low. This phenomenon is attributed to the extensive shared variations among species caused by incomplete lineage sorting and widespread hybridization (Svardal et al., 2021). Another study also demonstrated that mean sequence divergence between Malawi and Victoria is approximately 0.76%, and when subtracting within-species diversity, the divergence reduces to 0.62% (Svardal et al., 2020). One of the revelations brought about by the boom in evolutionary genomics over the last decade has been that hybridization between closely related animal species is the rule rather than the exception (Mallet et al., 2016; Novikova et al., 2016; Svardal et al., 2017). This is particularly true for young evolutionary radiations (Lamichhaney et al., 2015; Stryjewski and Sorenson, 2017; Edelman et al., 2019). López-Fernández et al. (2005), Lopez-Fernández et al. (2010) documented adaptive radiations in Geophagini and Heroni involving ecomorphological specializations, life history diversification, and rapid divergence. Aquarium experiments suggests that hybridization is in principle possible between thousands of haplochromine cichlid species whenever they come into contact. Therefore, the high mitogenome similarity between *A. citrinellus* and *A. amarillo* (*S. discus* and *S. haraldi*; *C. monoculus* and *C. ocellaris*) observed in this study resulted from interspecific hybridization, rather than synonymy or taxonomic misidentification.

## 5 Conclusion

The present study reports the complete mitogenome of *B. cupido.* Analysis of structural features and sequence variation across protein-coding and non-coding genes provides significant insights into the mitogenomic evolution of this species compared to other members within the tribe Geophagini. (Geophaginae). Phylogenetic analyses of the five major tribes of New World cichlids revealed that Geophagini, Cichlasomatini (Cichlasomatinae), and Cichlini (Cichlinae) were monophyletic, whereas Astronotini (Astronotinae) remained polyphyletic, suggesting a need for further taxonomic revision within this clade. Notably, the high degree of mitogenomic similarity observed among species within the same genus in this study appears to stem from historical or ongoing interspecific hybridization, rather than taxonomic misidentification or synonymy. Overall, this work presents the first fully sequenced mitochondrial genome for *B. cupido*, which serves as a foundational resource for future studies on its evolutionary history, population genetics, and phylogenetic relationships within the diverse Cichlidae family.

## Data Availability

The datasets presented in this study can be found in online repositories. The names of the repository/repositories and accession number(s) can be found below: https://www.ncbi.nlm.nih.gov/genbank/, OP595705.

## Abbreviations

BI, Bayesian inference; ML, Maximum likelihood; NCBI, National Center for Biotechnology Information; RSCU, Relative synonymous codon usage.

## References

[B1] Adrian-Kalchhauser I. Svensson O. Kutschera V. E. Alm Rosenblad M. Pippel M. Winkler S. (2017). The mitochondrial genome sequences of the round goby and the sand goby reveal patterns of recent evolution in gobiid fish. BMC Genomics 18, 177. 10.1186/s12864-017-3550-8 28209125 PMC5314710

[B2] Bernt M. Donath A. Jühling F. Externbrink F. Florentz C. Fritzsch G. (2013). MITOS: improved *de novo* metazoan mitochondrial genome annotation. Mol. Phylogenet. Evol. 69, 313–319. 10.1016/j.ympev.2012.08.023 22982435

[B3] Bibb M. J. Van Etten R. A. Wright C. T. Walberg M. W. Clayton D. A. (1981). Sequence and gene organization of Mouse mitochondrial DNA. Cell 26, 167–180. 10.1016/0092-8674(81)90300-7 7332926

[B4] Blanco-Bercial L. Bradford-Grieve J. Bucklin A. (2011). Molecular phylogeny of the Calanoida (crustacea: copepoda). Mol. Phylogenet. Evol. 59, 103–113. 10.1016/j.ympev.2011.01.008 21281724

[B5] Cichocki F. P. (1976). Cladistic history of cichild fishes and reproductive strategies of the American genera Amrichthys, biotodoma and geophagw. Ann Arbor: The University of Michigan, 705. Ph.D. Thesis.

[B6] Cichocki F. P. (1977). Tidal cycling and parental behavior of the cichlid fish, biotodoma cupido. Environ. Biol. Fishes 1, 159–169. 10.1007/bf00000407

[B7] Colatreli O. P. Meliciano N. V. Toffoli D. Farias I. P. Hrbek T. (2012). Deep phylogenetic divergence and lack of taxonomic concordance in species of astronotus (cichlidae). Int. J. Evol. Biol. 2012, 915265–915268. 10.1155/2012/915265 22779032 PMC3388587

[B8] Concheiro-Pérez G. A. Říčan O. Ortí G. Bermingham E. Doadrio I. Zardoya R. (2007). Phylogeny and biogeography of 91 species of heroine cichlids (teleostei: cichlidae) based on sequences of the cytochrome b gene. Mol. Phylogenetics Evol. 43, 91–110. 10.1016/j.ympev.2006.08.012 17045493

[B9] Dierckxsens N. Mardulyn P. Smits G. (2017). NOVOPlasty: *de novo* assembly of organelle genomes from whole genome data. Nucleic Acids Res. 45, e18. 10.1093/nar/gkw955 28204566 PMC5389512

[B10] Edelman N. B. Frandsen P. B. Miyagi M. Clavijo B. Davey J. Dikow R. B. (2019). Genomic architecture and introgression shape a butterfly radiation. Science 366, 594–599. 10.1126/science.aaw2090 31672890 PMC7197882

[B11] Gissi C. Iannelli F. Pesole G. (2008). Evolution of the mitochondrial genome of metazoa as exemplified by comparison of congeneric species. Heredity 101, 301–320. 10.1038/hdy.2008.62 18612321

[B12] Jondeung A. Sangthong P. Zardoya R. (2007). The complete mitochondrial DNA sequence of the mekong giant catfish (pangasianodon gigas), and the phylogenetic relationships among Siluriformes. Gene 387, 49–57. 10.1016/j.gene.2006.08.001 17067766

[B13] Kalyaanamoorthy S. Minh B. Q. Wong T. K. F. von Haeseler A. Jermiin L. S. (2017). ModelFinder: fast model selection for accurate phylogenetic estimates. Nat. Methods 14, 587–589. 10.1038/nmeth.4285 28481363 PMC5453245

[B14] Katoh K. Standley D. M. (2013). MAFFT multiple sequence alignment software version 7: improvements in performance and usability. Mol. Biol. Evol. 30, 772–780. 10.1093/molbev/mst010 23329690 PMC3603318

[B15] Ki J. S. Park H. G. Lee J. S. (2009). The complete mitochondrial genome of the cyclopoid copepod paracyclopina nana: a highly divergent genome with novel gene order and atypical gene numbers. Gene 435, 13–22. 10.1016/j.gene.2009.01.005 19393182

[B16] Kullander S. O. Norén M. Friðriksson G. B. Santos de Lucena C. A. (2010). Phylogenetic relationships of species of crenicichla (teleostei: cichlidae) from southern South America based on the mitochondrial cytochrome b gene. J. Zoological Syst. Evol. Res. 48 (3), 248–258.

[B17] Kumar S. Tamura K. Nei M. (1994). MEGA: molecular evolutionary genetics analysis software for microcomputers. Comput. Appl. Biosci. 10, 189–191. 10.1093/bioinformatics/10.2.189 8019868

[B18] Kundu S. De Alwis P. Kim A. Lee S. Kang H.-E. Go Y. (2023). Mitogenomic characterization of Cameroonian endemic Coptodon camerunensis (cichliformes: cichlidae) and matrilineal phylogeny of old-world cichlids. Genes 14, 1591. 10.3390/genes14081591 37628642 PMC10454717

[B19] Lamichhaney S. Berglund J. Almén M. S. Maqbool K. Grabherr M. Martinez-Barrio A. (2015). Evolution of Darwin’s finches and their beaks revealed by genome sequencing. Nature 518, 371–375. 10.1038/nature14181 25686609

[B20] Leibel W. S. (1995). Electrophoretic analysis of LDH phenotype supports splitting of the genus geophagus sensu gosse (teleostei, cichlidae). Copeia 1995, 217–223. 10.2307/1446819

[B21] Letunic I. Bork P. (2021). Interactive tree of Life (iTOL) v5: an online tool for phylogenetic tree display and annotation. Nucleic Acids Res. 49, 293–296. 10.1093/nar/gkab301 33885785 PMC8265157

[B22] Lopez-Fernández H. Honeycutt R. L. Winemiller K. O. (2005). Molecular phylogeny and evidence for an adaptive radiation of geophagine cichlids from South America (perciformes: labroidei). Mol. Phylogenetics Evol. 34, 227–244. 10.1016/j.ympev.2004.09.004 15579395

[B23] Lopez-Fernández H. Winemiller K. O. Honeycutt R. L. (2010). Multilocus phylogeny and rapid radiations in Neotropical cichlid fishes (perciformes: cichlidae: cichlinae). Mol. Phylogenetics Evol. 55, 1070–1086. 10.1016/j.ympev.2010.02.020 20178851

[B24] Lowe T. M. Eddy S. R. (1997). TRNAscan-SE: a program for improved detection of transfer RNA genes in genomic sequence. Nucleic Acids Res. 25, 955–964. 10.1093/nar/25.5.955 9023104 PMC146525

[B25] Mallet J. Besansky N. Hahn M. W. (2016). How reticulated are species? BioEssays 38, 140–149. 10.1002/bies.201500149 26709836 PMC4813508

[B26] Mattos J. L. O. Costa W. J. E. M. (2018). Three new species of the “geophagus” brasiliensis species group from the northeast Brazil (cichlidae, geophagini). Zoosystematics Evol. 94, 325–337. 10.3897/zse.94.22685

[B27] Minh B. Q. Schmidt H. A. Chernomor O. Schrempf D. Woodhams M. D. von Haeseler A. (2020). IQ-TREE 2: new models and efficient methods for phylogenetic inference in the genomic era. Mol. Biol. Evol. 37, 1530–1534. 10.1093/molbev/msaa015 32011700 PMC7182206

[B28] Morey G. A. M. Arimuya M. V. Boeger W. A. Monogenoidea N. (2019). Biotodomella mirospinata gen. Nov., sp. Zool. (Curitiba) 36. 10.3897/zoologia.36.e38455

[B29] Morey G. A. M. Dávila H. A. Arimuya M. V. de Sousa A. L. Cruces C. L. Chero J. D. (2024). Three new species of sciadicleithrum (monogenoidea, dactylogyridae) parasitizing cichlid fishes (cichliformes: cichlidae) in the Northeastern Peru. Acta Parasitol. 69 (3), 1674–1681. 10.1007/s11686-024-00895-y 39167260

[B30] Musilová Z. Rícan O. Janko K. Novák J. (2008). Molecular phylogeny and biogeography of the Neotropical cichlid fish tribe Cichlasomatini (Teleostei: cichlidae: cichlasomatinae). Mol. Phylogenetics Evol. 46, 659–672. 10.1016/j.ympev.2007.10.011 18055225

[B31] Nakayama Y. Yamaguchi H. Einaga N. Esumi M. (2016). Pitfalls of DNA quantification using DNA-binding fluorescent dyes and suggested solutions. PLOS One 11, e0150528. 10.1371/journal.pone.0150528 26937682 PMC4777359

[B32] Novikova P. Y. Hohmann N. Nizhynska V. Tsuchimatsu T. Ali J. Muir G. (2016). Sequencing of the genus Arabidopsis identifies a complex history of nonbifurcating speciation and abundant trans-specific polymorphism. Nat. Genet. 48, 1077–1082. 10.1038/ng.3617 27428747

[B33] Ottoni F. P. Mattos J. L. O. (2015). Phylogenetic position and redescription of the endangered cichlid Nannacara hoehnei, and description of a new genus from Brazilian Cerrado (Teleostei, cichlidae, cichlasomatini). Vertebr. Zool. 65, 65–79. 10.3897/vz.65.e31507

[B34] Perna N. T. Kocher T. D. (1995). Patterns of nucleotide composition at fourfold degenerate sites of animal mitochondrial genomes. J. Mol. Evol. 41, 353–358. 10.1007/BF00186547 7563121

[B35] Piálek L. Rícan O. Casciotta J. Almiron A. Zrzavý J. (2012). Multi locus phylogeny of crenicichla (teleostei: cichlidae), with biogeography of the C. lacustris group: species flocks as a model for sympatric speciation in Rivers. Mol. Phylogenetics Evol. 62, 46–61. 10.1016/j.ympev.2011.09.006 21971056

[B36] Porebski S. Bailey L. G. Baum B. R. (1997). Modification of a CTAB DNA extraction protocol for plants containing high polysaccharide and polyphenol components. Plant Mol. Biol. Rep. 15, 8–15. 10.1007/BF02772108

[B37] Ranwez V. Douzery E. J. P. Cambon C. Chantret N. Delsuc F. Macse V. (2018). MACSE v2: toolkit for the alignment of coding sequences accounting for frameshifts and stop codons. Mol. Biol. Evol. 35, 2582–2584. 10.1093/molbev/msy159 30165589 PMC6188553

[B38] Reiss H. Hoarau G. Dickey‐Collas M. Wolff W. J. (2009). Genetic population structure of marine fish: mismatch between biological and fisheries management units. Fish 10, 361–395. 10.1111/j.1467-2979.2008.00324.x

[B39] Ronquist F. Teslenko M. van der Mark P. Ayres D. L. Darling A. Höhna S. (2012). MrBayes 3.2: efficient bayesian phylogenetic inference and model choice across a large model space. Syst. Biol. 61, 539–542. 10.1093/sysbio/sys029 22357727 PMC3329765

[B40] Stryjewski K. F. Sorenson M. D. (2017). Mosaic genome evolution in a recent and rapid avian radiation. Nat.Ecol. Evol. 2017 (1), 1912–1922. 10.1038/s41559-017-0364-7 29085063

[B41] Svardal H. Jasinska A. J. Apetrei C. Coppola G. Huang Y. Schmitt C. A. (2017). Ancient hybridization and strong adaptation to viruses across African vervet monkey populations. Nat. Genet. 2017, 1705–1713. 10.1038/ng.3980 29083404 PMC5709169

[B42] Svardal H. Quah F. X. Malinsky M. Ngatunga B. P. Miska E. A. Salzburger W. (2020). Ancestral hybridization facilitated species diversification in the Lake Malawi cichlid fish adaptive radiation. Mol. Biol. Evol. 37, 1100–1113. 10.1093/molbev/msz294 31821500 PMC7086168

[B43] Svardal H. Salzburger W. Malinsky M. (2021). Genetic variation and hybridization in evolutionary radiations of cichlid fishes. Annu. Rev. Animal Biosci. 9, 55–79. 10.1146/annurev-animal-061220-023129 33197206

[B44] Tougard C. Davila C. R. G. Römer U. Duponchelle F. Cerqueira F. Paradis E. (2017). Tempo and rates of diversification in the south American cichlid genus apistogramma (teleostei: perciformes: cichlidae). PLoS One 12, e0182618. 10.1371/journal.pone.0182618 28873089 PMC5584756

[B45] Wang J. Tai J. Zhang W. He K. Lan H. Liu H. (2023). Comparison of seven complete mitochondrial genomes from lamprologus and neolamprologus (chordata, teleostei, Perciformes) and the phylogenetic implications for cichlidae. ZooKeys 1184, 115–132. 10.3897/zookeys.1184.107091 38314327 PMC10838552

[B46] Ye W. Zhao X. Xu T. Wang J. Liu H. (2023). Complete mitochondrial genomes of Lycosa grahami and lycosa sp. (araneae: lycosidae): comparison within the family lycosidae. Int. J. Trop. Insect Sci. 43, 533–545. 10.1007/s42690-023-00965-0

[B47] Yu J. N. Kwak M. (2015). The complete mitochondrial genome of Brachymystax lenok tsinlingensis (salmoninae, salmonidae) and its intraspecific variation. Gene 573, 246–253. 10.1016/j.gene.2015.07.049 26188159

[B48] Zhang D. Gao F. Jakovlić I. Zou H. Zhang J. Li W. X. (2020). PhyloSuite: an integrated and scalable desktop platform for streamlined molecular sequence data management and evolutionary phylogenetics studies. Mol. Ecol. Resour. 20, 348–355. 10.1111/1755-0998.13096 31599058

[B49] Zhu T. Sato Y. Sado T. Miya M. Iwasaki W. MitoFish M. (2023). MitoFish, MitoAnnotator, and MiFish pipeline: updates in 10 years. Mol. Biol. Evol. 40, msad035. 10.1093/molbev/msad035 36857197 PMC9989731

