# Supplementary material for: The first complete mitochondrial genome of Biotodoma cupido (Cichiliformes: Cichlidae) and its phylogeny
Source: Front Genet. 2025 Aug 29;16:1623517. doi: 10.3389/fgene.2025.1623517 (PMC12425952; doi:10.3389/fgene.2025.1623517)
Supplement: Supplementary file 1 [file DataSheet1.pdf]

Supplementary Information 1

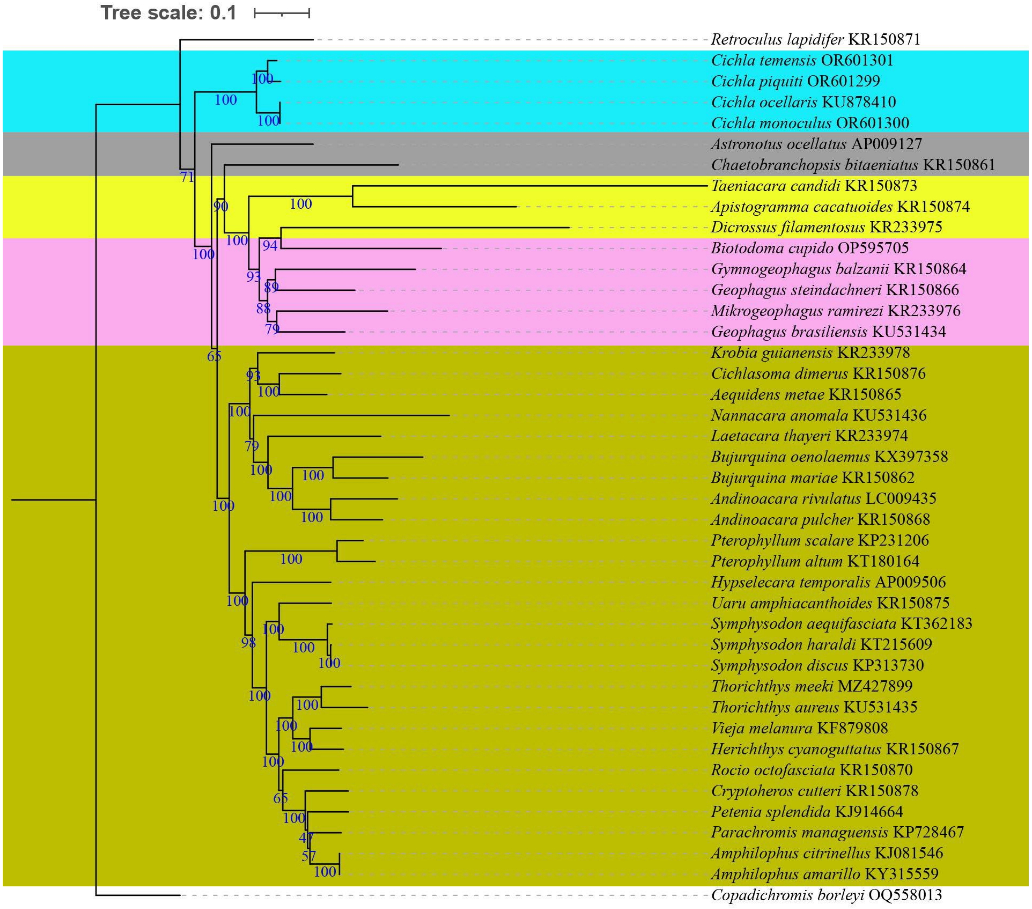

Figure S1. Molecular phylogenetic tree constructed based on maximum likelihood analysis. Numbers at nodes represent the bootstrap support in the ML analysis. In the constructed phylogenetic tree, distinct colors represent different species clusters: brownish yellow for tribe Cichlasomatinae, light blue for tribe Cichlinae, pink and yellow for tribe Geophaginae, and grey for tribe Astronotinae.

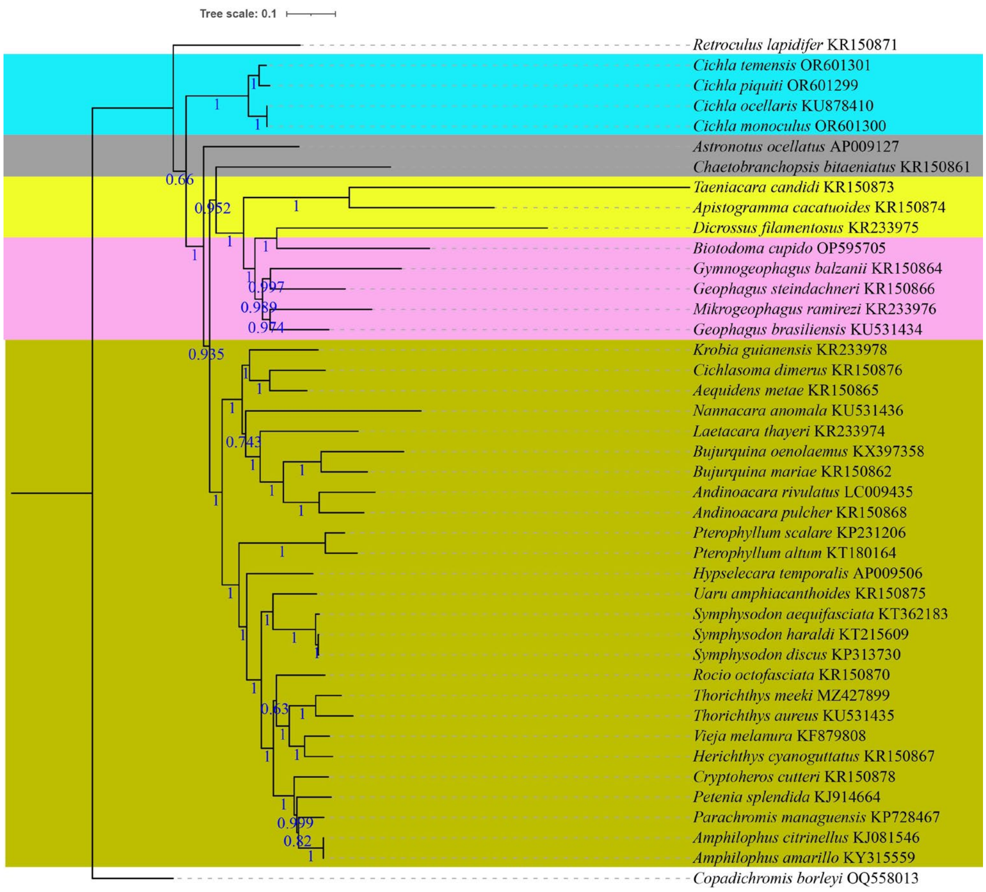

Figure S2. Molecular phylogenetic tree constructed based on Bayesian inference analysis. Numbers at nodes represent the Bayesian posterior probability in the BI analysis. In the constructed phylogenetic tree, distinct colors represent different species clusters: brownish yellow for tribe Cichlasomatinae, light blue for tribe Cichlinae, pink and yellow for tribe Geophaginae, and grey for tribe Astronotinae.
